# Fluorescence labeling of a Na_V_1.7-targeted peptide for near-infrared nerve visualization

**DOI:** 10.1186/s13550-020-00630-4

**Published:** 2020-05-14

**Authors:** Junior Gonzales, Giacomo Pirovano, Chun Yuen Chow, Paula Demetrio de Souza Franca, Lukas M. Carter, Julie K. Klint, Navjot Guru, Jason S. Lewis, Glenn F. King, Thomas Reiner

**Affiliations:** 1 Department of Radiology, Memorial Sloan Kettering Cancer Center, 1275 York Avenue, New York, NY 10065 USA; 2 Institute for Molecular Bioscience, The University of Queensland, St. Lucia, Queensland 4072 Australia; 3 Current address: H. Lundbeck A/S, Ottiliavej 9, 2500 Valby, Denmark; 4 Center for Molecular Imaging and Nanotechnology (CMINT), Memorial Sloan Kettering Cancer Center, New York, NY 10065 USA; 5 Department of Radiology, Weill Cornell Medical College, 1300 York Avenue, New York, NY 10065 USA; 6 Molecular Pharmacology Program, Memorial Sloan Kettering Cancer Center, New York, NY 10065 USA; 7 Department of Pharmacology, Weill-Cornell Medical College, New York, NY 10065 USA; 8 Chemical Biology Program, Memorial Sloan Kettering Cancer Center, 1275 York Avenue, New York, NY 10065 USA

**Keywords:** Hs1a-FL, Nerve imaging, Near-infrared imaging, Intraoperative

## Abstract

**Background:**

Accidental peripheral nerve injury during surgical intervention results in a broad spectrum of potentially debilitating side effects. Tissue distortion and poor visibility can significantly increase the risk of nerve injury with long-lasting consequences for the patient. We developed and characterized Hs1a-FL, a fluorescent near-infrared molecule for nerve visualization in the operating theater with the aim of helping physicians to visualize nerves during surgery. Hs1a was derived from the venom of the Chinese bird spider, *Haplopelma schmidti*, and conjugated to Cy7.5 dye. Hs1a-FL was injected intravenously in mice, and harvested nerves were imaged microscopically and with epifluorescence.

**Results:**

Hs1a-FL showed specific and stable binding to the sodium channel Na_V_1.7, present on the surface of human and mouse nerves. Hs1a-FL allowed epifluorescence visualization of sciatic mouse nerves with favorable nerve-to-muscle contrast.

**Conclusions:**

Fluorescent Na_V_1.7-targeted tracers have the potential to be adopted clinically for the intraoperative visualization of peripheral nerves during surgery, providing guidance for the surgeon and potentially improving the standard of care.

## Background

Unintentional resection or injury of nerves during medical interventions is a significant concern during surgery [1, 2]. During surgery, there is always a degree of risk for nerves to be cut, crushed, tied off, penetrated and twisted by screws, or even injured during the removal of devices. In addition, they can be stretched by retractors, cut, or thermally damaged by electric knife, hardening bone cement, or during coagulation [3, 4]. These unintentional, iatrogenic complications typically occur because nerves are not clearly visible to the surgeon or could be mistaken for a vessel or tendon. Certain surgical procedures are considered high risk for resulting in nerve injury. These include, but are not limited to, osteosynthesis and osteotomy, arthrodesis, lymph node biopsy in the neck, parotidectomies, thyroid surgeries, carpal tunnel syndrome surgery, varicose vein surgery, excision of Baker cysts, and inguinal herniorrhaphies.

For example, in head and neck surgery, iatrogenic nerve injuries can result in facial paralysis, hoarseness or weakening of voice, respiratory distress, and other neurological complications with harsh lifestyle implications for the patient [5–8]. As a result, one out of four patients with neuropathic pain identifies surgical morbidity as the originating cause [9]. Oncologic surgery, in particular, poses a risk of nerve injury as anatomy is often distorted by the disease [10–17]. There are existing tools for preoperative and perioperative nerve enhancement [18–20], and they mostly rely on magnetic resonance imaging (MRI) and nerve ultrasound [21, 22]. However, fluorescent nerve imaging agents [23, 24], as well as multimodal optical imaging techniques, are raising interest for intraoperative applications [25–35]. However, the availability of an intraoperative imaging agent that could clearly identify small nerve branches or even larger trunks below the tissue surface, especially in cases of reoperation when normal anatomy is disrupted, could significantly improve surgical precision. In an attempt to respond to this need, we developed Hs1a-FL, a near-infrared imaging agent that could be used in the described surgical settings.

**Fig. 1:**
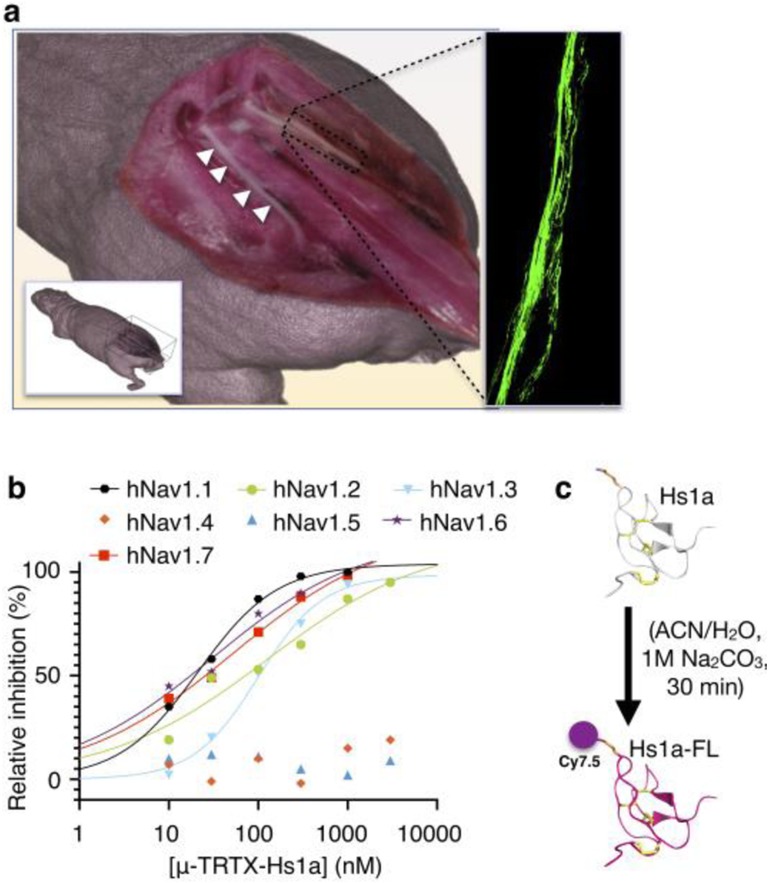
Ion channel selectivity and chemical synthesis of Hs1a-FL. **a** Representative view of the experimental settings. A 3D rendering of a frozen and sliced mouse. White arrows show the left sciatic nerve. Right sciatic nerve magnification shows the fluorescent Hs1a-FL agent bound to the nerve surface. **b** Selectivity of Hs1a towards human Na_V_ channels stably expressed in HEK293 cells. Calculated IC_50_ values were hNa_V_1.1; 19.4 nM, hNa_V_1.2; 81.2 nM, hNa_V_1.3; 106.8 nM, hNa_V_1.4; > 3000 nM, hNa_V_1.5; > 3000 nM, hNa_V_1.6; 19.2 nM, hNa_V_1.7; 26.9 nM. Each point on the curve is an average of 3–11 cells. **c** Reaction scheme for conjugation of Hs1a peptide with Cyanine7.5-NHS ester dye. The ribbon model of Hs1a-FL shows disulfide bridges (in yellow) and shows the attachment of one dye to the peptide (orange/blue)

Recombinant peptide Hs1a was derived from the venom of the Chinese bird spider, *Haplopelma schmidti*. Hs1a proved to be a potent and subtype-selective inhibitor of sodium channel Na_V_1.7, a key signal-transmitter located on nerve surfaces [36]. Our fluorescently labeled version of Hs1a targets Na_V_1.7 receptors and has the potential to be used as a vector for delivering an optical sensor to peripheral nerves in vivo. We show that the labeling of Hs1a with Cy7.5 *N*-hydroxy succinimide (NHS) ester could be used as a practical tool for nerve visualization during surgery in a preclinical mouse model (Fig. 1a). We anticipate that this technology will have the potential to directly impact the surgical standard of care by lending contrast to nerves, thereby decreasing iatrogenic injury and therefore surgical morbidity.

## Methods

### General

Unless otherwise stated, all solvents and reagents were obtained from Sigma-Aldrich or Fisher Scientific and were used without further purification. Cyanine7.5 (Cy7.5) was purchased from Lumiprobe (MD, USA). Anti-Na_V_1.7 antibody [N68/6] was purchased from Abcam (ab85015). Water (> 18.2 MΩ cm at 25 °C) was obtained from an Alpha-Q Ultrapure water system (Millipore). Acetonitrile (AcN) was of high-performance liquid chromatography (HPLC) grade and was purchased from Fisher Scientific. Phosphate-buffered saline (PBS) without Ca^2+^ or Mg^2+^ was obtained from the Media Preparation Facility at Memorial Sloan Kettering Cancer Center (MSKCC) and used for all in vivo injections. Reverse-phase (RP) HPLC purifications were performed on a Shimadzu HPLC system equipped with a DGU-20A degasser, SPD-M20A UV detector, LC-20AB pump system, and a CBM-20A communication bus module using RP-HPLC columns (Atlantis T3 C18, 5 μm, 4.6 × 250 mm, P/N: 186003748). Epifluorescence imaging was performed on an IVIS Spectrum imaging system (PerkinElmer). Confocal microscopy images were captured using a Leica SP8 inverted-stand confocal microscope equipped with a tunable white light laser that ranges from 470 to 670 nm. The microscope is also equipped with a 405-nm diode, argon laser (with 476 nm, 488 nm, 496 nm, and 514 nm laser line), and a 725-nm laser for near infra-red NIR imaging coupled with avalanche photo-diode detectors (APDs) which were used for detection of Hs1a-FL.

### Synthesis of Hs1a

Recombinant Hs1a was produced via expression in the periplasm of *E. coli* using a protocol optimized for production of disulfide-rich peptides [37]. The recombinant peptide containing a non-native N-terminal glycine residue was purified by nickel affinity chromatography after liberation from the His_6_-MBP fusion tag via cleavage with tobacco etch virus protease. LC-ESI-MS (ES+), m/z calculated for [C_164_H_251_N_49_O_47_S_6_] 3850.74, [C_164_H_251_N_49_O_47_S_6_ + 3H]^3+^ 1284.58, found [M + 3H]^3+^ 1285.00, [C_164_H_251_N_49_O_47_S_6_ + 4H]^4+^ 963.69, found [M + 4H]^4+^ 964.20, [C_164_H_251_N_49_O_47_S_6_ + 5H]^5+^ 771.15, found [M + 5H]^5+^ 772.60, [C_164_H_251_N_49_O_47_S_6_ + 6H]^6+^ 642.79, found 643.25.

### Synthesis of Hs1a-FL

Hs1a was discovered in a high-throughput fluorescent-based assay to screen spider venoms against hNa_V_1.7, as previously described [38]. Recombinant Hs1a peptide (0.26 mM, 200 μg in 200 μL of AcN) and Na_2_CO_3_ (1 M, 40 μL) were transferred into a 3-mL amber vial with a magnetic bar stirrer. Cy7.5-NHS (4 μL of a 24 mM solution) was dissolved in AcN and added dropwise to the reaction mixture. The final volume of the reaction mixture was 350 μL. The reaction mixture was stirred for at least 10 min before dilution with 100 μL of water. This reaction produced mono- and di-adducts of Cy7.5, which were purified and separated using RP-HPLC. Fractions containing the mono-adduct of Hs1a-FL were concentrated; then, the solvent was removed in vacuo to afford a dark greenish powder (20 μg, 14% yield from Hs1a peptide). This purified compound was then formulated in 100% Ca^2+^/Mg^2+^-free PBS or 10% dimethyl sulfoxide (DMSO) and PBS. LC-ESI-MS (ES+), m/z calculated for [C_209_H_298_N_51_O_48_S_6_] 4482.12, [C_209_H_298_N_51_O_48_S_6_ + 3H]^3+^ 1495.04, found [M + 3H]^3+^ 1495.45, [C_209_H_298_N_51_O_48_S_6_ + 4H]^4+^ 1121.53, found [M + 4H]^4+^ 1121.75, [C_209_H_298_N_51_O_48_S_6_ + 5H]^5+^ 897.42, found [M + 5H]^5+^ 897.75, [C_209_H_298_N_51_O_48_S_6_ + 6H]^6+^ 748.02, found [M + 6H]^6+^ 748.25

### Cell lines

HEK293 cells stably expressing the human Na_V_ channel β1 subunit (hNa_V_β1) in combination with the α subunit hNa_V_1.1, hNa_V_1.2, hNa_V_1.3, hNa_V_1.4, hNa_V_1.5, hNa_V_1.6, or hNa_V_1.7 (Scottish Biomedical, Glasgow, UK) were cultured in DMEM/F-12 media (1:1), supplemented with 10% fetal bovine serum, 400 mg/mL geneticin, and 100 mM non-essential amino acids (all reagents from Invitrogen) at 37 °C and in 5% CO_2_.

### Electrophysiology

Whole-cell patch-clamp experiments were performed at room temperature using a QPatch 16x automated electrophysiology platform (Sophion Bioscience, Denmark) using 16-channel planar patch-chip plates (QPlates) with a patch-hole diameter of 1 μm and resistance of 2 MΩ. Whole-cell currents were filtered at 5 kHz (8-pole Bessel) and digitized at 25 kHz. A P4 online leak-subtraction protocol was used with non-leak-subtracted currents acquired in parallel. The extracellular solution was 2 mM CaCl_2_, 1 mM MgCl_2_, 10 mM HEPES, 4 mM KCl, and 145 mM NaCl at pH 7.4, and the intracellular solution was 140 mM CsF, 1 mM/5 mM EGTA/CsOH, 10 mM HEPES, and 10 mM NaCl at pH 7.3. Hs1a-FL were dissolved in extracellular solution with 0.1% bovine serum albumin (BSA). Concentration-response data were obtained using five concentrations of peptide (2 nM to 10 μM). HEK293-hNa_V_ cells were clamped at a holding potential of − 60 mV for Na_V_1.1, − 65 mV for Na_V_1.2, − 60 mV for Na_V_1.3, − 75 mV for Na_V_1.4, − 105 mV for Na_V_1.5, − 60 mV for Na_V_1.6, and − 75 mV for Na_V_1.7. For each concentration, 10 μL of peptide was added for 6 s before applying the following voltage protocol: − 80 mV for 10 ms, − 120 mV for 200 ms, 0 mV for 20 ms, then return to − 80 mV potential. This was repeated once every 60 s during liquid applications. Cells were otherwise held at the holding potential when the above voltage protocol was not executed. Upon establishment of the whole cell recording configuration, a total of five applications of the extracellular solution (1× control buffer, 3× test compound/control, 1 μM tetrodotoxin (TTX; positive control)), all containing 0.1% BSA (except for the TTX solution) were made on each cell. The voltage protocol was executed 10 times after each application. Currents were sampled at 25 kHz and filtered at 5 kHz with an 8-pole Bessel filter. The series resistance compensation level was set at 80%. All experiments were performed at room temperature (~ 22 °C). IC_50_ values were determined from non-linear regression of concentration-response data using GraphPad Prism.

### Animal studies

Female athymic nude mice (4–8 weeks old, athymic-nude (outbred) (stock#:088; Envigo, USA) were allowed to acclimatize at the MSKCC vivarium for 1 week with ad libitum food and water prior to the experimental procedure. For imaging experiments, animals were sacrificed 30 min post-tail vein injection of Hs1a-FL, Hs1a/Hs1a-FL, or PBS. All animal experiments were performed in accordance with institutional guidelines and approved by the MSKCC Institutional Animal Care and Use Committee, following the NIH guidelines for animal welfare.

### Mouse cryosectioning and image-based reconstruction

Post-euthanasia, a representative mouse was fast frozen in hexanes with dry ice. Coronal cryosectioning and white-light imaging were performed by EMIT using a Xerra imager; following each sequential removal of 50-μm-thick slices, the tissue-embedded block was imaged at 30 μm in-plane resolution. A 3D image volume of the mouse was generated through multiplanar reformation using 3D Slicer software for anatomic visualization (Fig. 1a).

### Immunohistochemistry

Immunohistochemical (IHC) staining experiments were used to detect the expression and abundance of sodium channel Na_V_1.7 in mouse sciatic nerve tissue. Anti-Na_V_1.7 antibody [N68/6] (Abcam ab85015) was found to specifically bind to mouse Na_V_1.7 (0.5 μg/mL). Paraffin-embedded formalin-fixed 5 μm sections were deparaffinized with EZPrep buffer. For IHC detection, a 3,3′-diaminobenzidine (DAB) detection kit (Ventana Medical Systems, Tucson, AZ) was used according to the manufacturer’s instructions. These experiments were performed at the MSKCC Molecular Cytology Core Facility using the Discovery XT processor (Ventana Medical System, Tucson, AZ). Adjacent sections were stained against IgG, to control for non-specific binding to Na_V_1.7. Sections were counterstained with hematoxylin and eosin (H&E) and coverslipped with Permount (Fisher Scientific, Pittsburgh, PA) for morphological evaluation of tissue characteristics.

### Confocal microscopy

For the confocal microscopy experiments, 5 μm cryosections of sciatic nerve tissues embedded in optimal cutting temperature compound (OCT) were used to determine the distribution and localization of Hs1a-FL from mice previously injected with the fluorescent agent Hs1a-FL (4 nmol, 45 μM of Hs1a-FL in 100 μL of PBS), the blocking solution Hs1a/Hs1a-FL (Hs1a-FL, 45 μM, 4 nmol and Hs1a 120 μM, 12 nmol in 100 μL PBS), or 100 μL of PBS. These resected nerves were incubated with Hoechst 33342 (20 μM, 1 nmol in 50 μL of PBS) to counterstain nuclei, which were subsequently embedded in Mowiol mounting medium. Fresh tissues were counterstained with Hoechst 33342 (20 μM, 1 nmol in 50 μL of PBS) and samples placed directly on a microscope slide for detection of the fluorescence signal of the fluorescent peptide.

### Epifluorescence imaging

One group of animals was intravenously injected with Hs1a-FL (4 nmol, 45 μM of Hs1a-FL in 100 μL of PBS, *n* = 3). A second group of animals was injected with Hs1a and Hs1a-FL (Hs1a-FL, 45 μM, 4 nmol and Hs1a, 120 μM, 12 nmol in 100 μL PBS, *n* = 3) or PBS (*n* = 3). Animals were sacrificed 30 min post-injection and epifluorescence images obtained. Epifluorescence images of the right sciatic nerve (RSN) and left sciatic nerve (LSN) were obtained in situ from all the mice in the study. Epifluorescence images of the biodistribution included RSN, LSN, muscle, heart, kidney, liver, and brain and were acquired with an IVIS Spectrum imaging system (PerkinElmer) using a predetermined filter set (excitation = 710/45 nm, emission = 800–820 nm). Autofluorescence was removed through spectral unmixing. Semiquantitative analysis of the Hs1a-FL signal was conducted by measuring the average radiant efficiency (in units of [p/s/cm^2^/sr]/[μW/cm^2^]) in regions of interest (ROIs) that were placed on all resected nerves and as well in all organs from the biodistribution under white light guidance.

### Statistical analyses

Statistical analyses were performed using GraphPad Prism 8. Unless otherwise stated, data points represent mean values, and error bars represent standard deviations of biological replicates. All *p* values were calculated using an unpaired *t* test. Statistical significance was considered for *p* values < 0.05 and as follows: ns = not significant, *p < 0.05, **p < 0.01, ***p < 0.001.

## Results

### Selectivity of Hs1a across Ion Channel Subtypes

Hs1a peptide was isolated from venom of the Chinese tarantula *Haplopelma schmidti*. To assess the selectivity across the various Na_v_ channel subtypes, Hs1a was tested on human Na_V_1.1–Na_V_1.7 channels stably expressed in HEK293 cells using automated patch-clamp techniques. Hs1a was shown to have inhibitory affinity for neuronal Na_v_ channels, with IC_50_ values in the low nanomolar range (19.4, 82, 107, 19.2, 26.9 nM for Na_V_1.1, Na_V_1.2, Na_V_1.3, Na_V_1.6, and Na_V_1.7, respectively), but it did not inhibit Na_V_1.4 and Na_V_1.5, which are found primarily in the muscle and heart, respectively at concentrations up to 3 μM (Fig. 1b, Table 1 in supplementary information).

### Design of the fluorescence peptide, Hs1a-FL

**Fig. 2:**
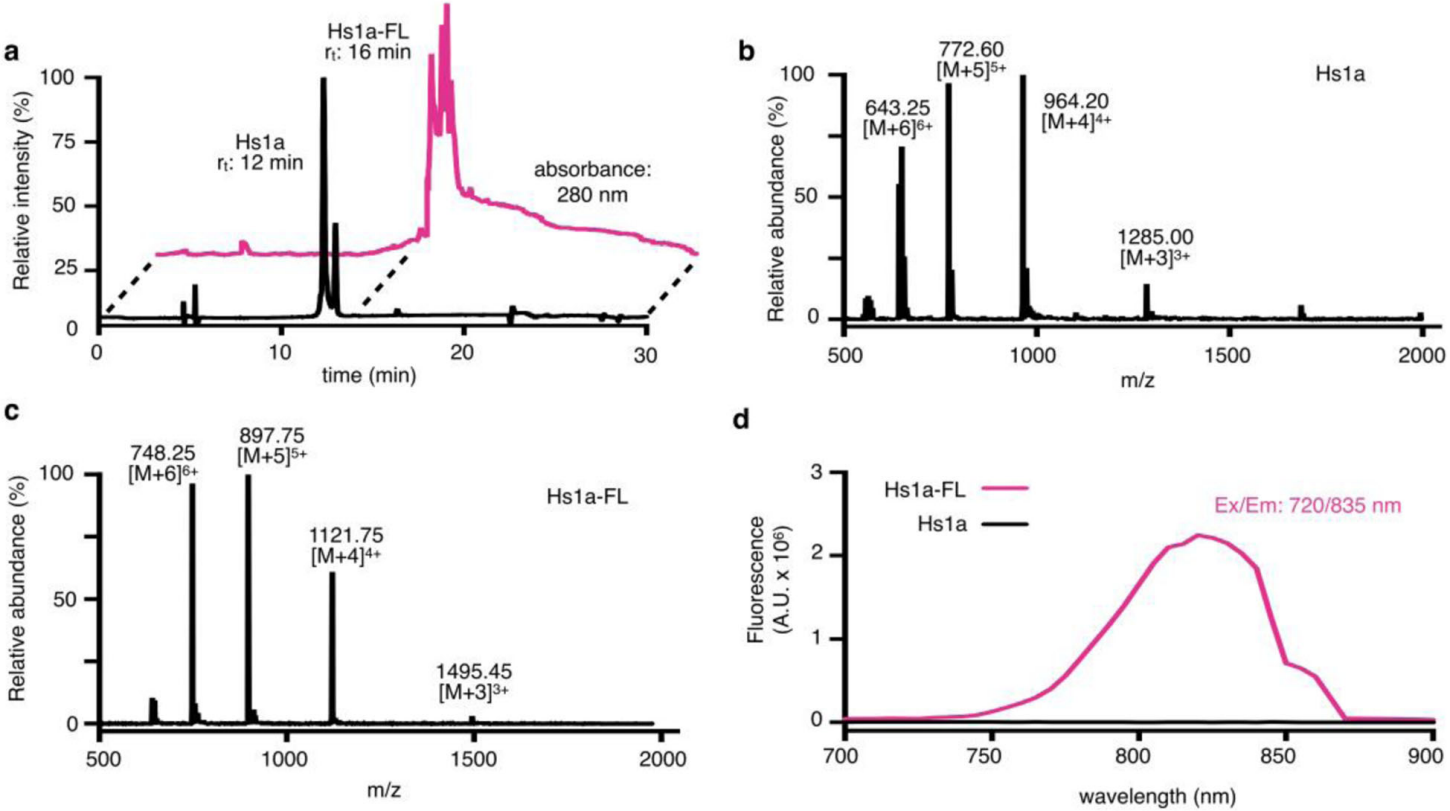
Chemical characterization of Hs1a-FL. **a** RP-HPLC chromatograms of Hs1a (black) and Hs1a-FL (pink) with absorbances observed at 280 nm. **b** LC-MS spectrum of Hs1a and **c** of Hs1a-FL. The mass spectra show four major ion species that correspond to the calculated mass of Hs1a peptide and four major ion species that confirm the calculated mass of Hs1a-FL after dye conjugation. **d** Fluorescence spectra (Ex/Em 720/835 nm) of 0.1 μM Hs1a peptide (black) and 0.1 μM Hs1a-FL (pink)

We used recombinant Hs1a to synthesize Hs1a-FL, a NIR-labeled version of Hs1a (Fig. 1c). We chose a NIR fluorophore for its emission wavelength with favorable tissue penetration potential for intraoperative applications. We modified Hs1a via nucleophilic substitution as previously described [30]. The synthesis was performed under basic conditions in a mixture of water and acetonitrile, with 14% yield. Retention time (*r*_t_) shifted from 12 min for the unmodified Hs1a to 16 min for Hs1a-FL (Fig. 2a and Fig. S1a). The major impurities were characterized as the partially reduced peptide, 3% (*r*_t_ 16.2 min), which was also present in the starting material (*r*_t_ 12 min, 80% and *r*_t_ 12.2 min, 20% for Hs1a and reduced Hs1a, respectively). LC/MS spectra for both Hs1a and Hs1a-FL showed clean peak families confirming the peptides’ calculated masses of 3850.74 Da and 4482.12 Da for Hs1a and Hs1a-FL, respectively (Fig. 2b, c, Table 2 in supplementary material). In addition, florescence of 0.1 μM Hs1a peptide and 0.1 μM Hs1a-FL were collected to confirm dye conjugation (Fig. 2d).

### Histology and Hs1a-FL imaging of mouse sciatic nerve

**Fig. 3:**
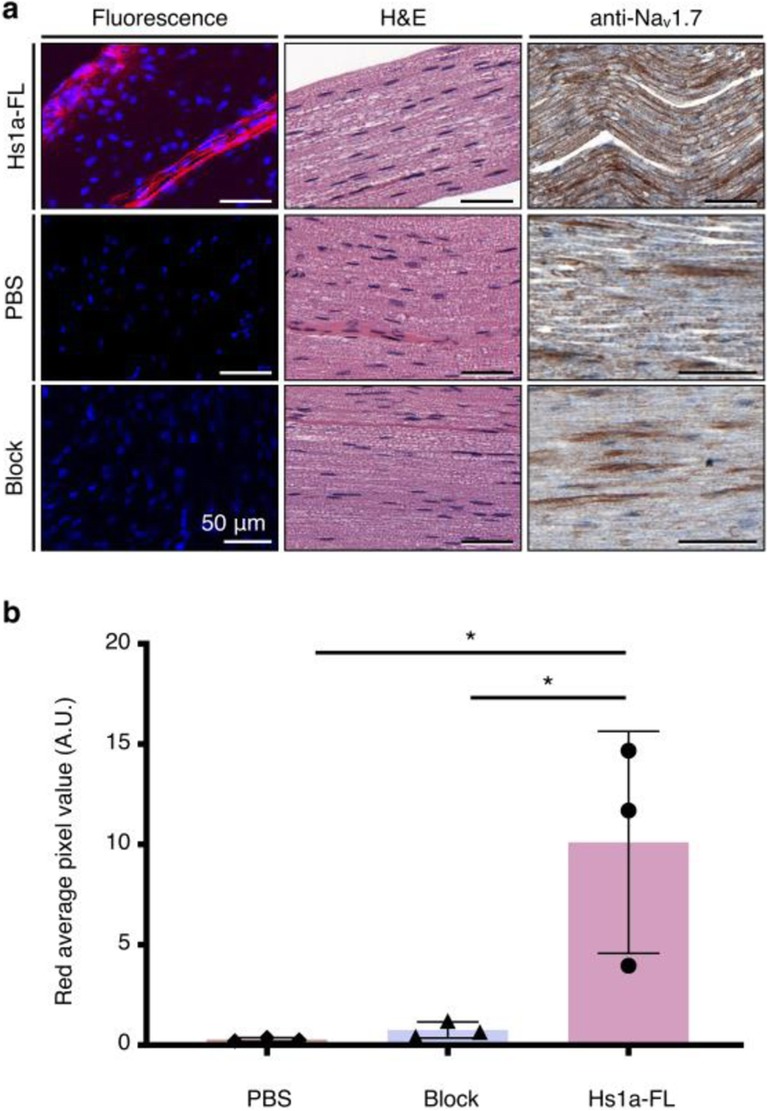
Ex vivo microscopy imaging of Hs1a-FL in mouse sciatic nerve. **a** Fluorescence of Hs1a-FL-stained mouse sciatic nerves compared to mice injected with vehicle (PBS) or co-injected with Hs1a (Hs1a-FL, 45 μM, 4 nmol and Hs1a 120 μM, 12 nmol in 100 μL PBS). H&E staining of adjacent nerve tissue and IHC staining, confirming the expression of Na_V_1.7. **b** Quantification of total detected fluorescence. Unpaired *t* test. *p value < 0.05

To assess the possibility of using Hs1a-FL to image sciatic nerves in vivo, mice were injected intravenously with Hs1a-FL alone (4 nmol, 45 μM of Hs1a-FL in 100 μL of PBS) or in combination with an excess of unmodified peptide (120 μM, 12 nmol in 100 μL PBS, block) and sacrificed 30 min after injection. Nerves were surgically harvested and flash-frozen in OCT blocks. Blocks were then sliced on a cryotome at a 10-μm thickness and imaged. Nerves were imaged to detect fluorescent signal and H&E stained to enable the visualization of Schwann cells within the nerve structure. Anti-Na_V_1.7 immunohistochemistry confirmed target availability (Fig. 3a). Confocal microscopy confirmed the presence of Hs1a-FL signal in injected mice. No signal was detected in mice injected with PBS or “blocking solution” containing Hs1a-FL and a 3-fold molar excess of unlabeled Hs1a (Fig. 3b). In addition, no staining was observed when using isotype control antibodies, confirming specificity (Fig. S1b).

### Ex vivo Hs1a-FL biodistribution

**Fig. 4:**
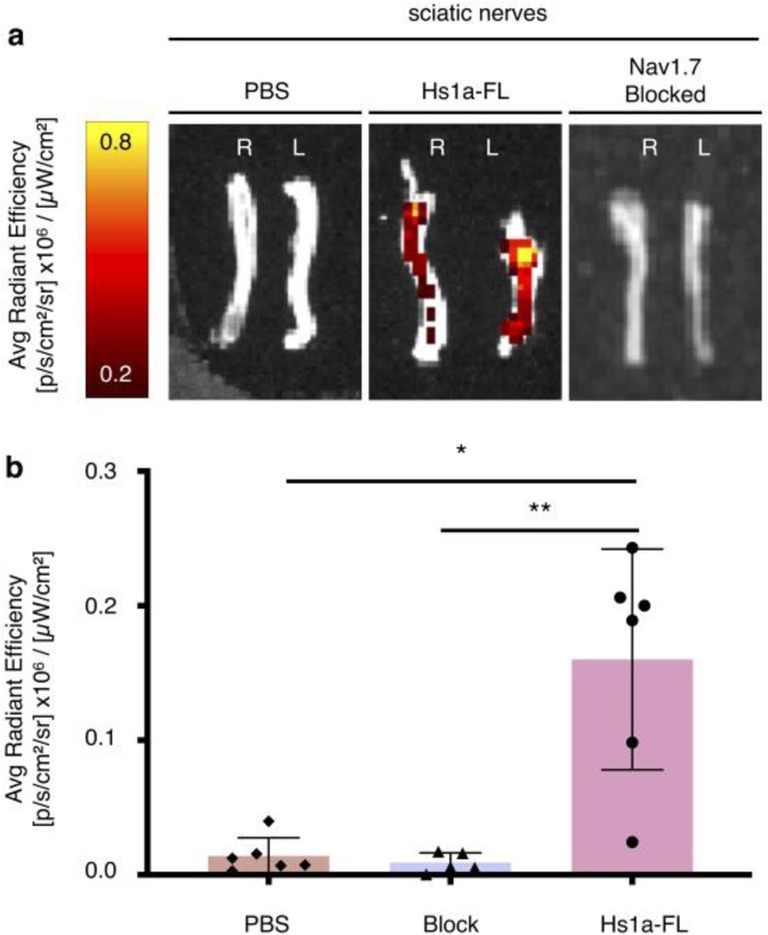
Epifluorescence imaging of fresh, unprocessed mouse sciatic nerves with Hs1a-FL. **a** Epifluorescence images of resected sciatic nerves from animals injected with PBS, Hs1a-FL (4 nmol, 45 μM of Hs1a-FL in 100 μL of PBS), and a Hs1a/Hs1a-FL mixture (Hs1a-FL, 45 μM, 4 nmol and Hs1a 120 μM, 12 nmol in 100 μL PBS). Images were taken 30 min after tail vein injection. **b** Fluorescence intensity quantification. Unpaired *t* test. *p value < 0.05; **p value < 0.01

Mice were injected intravenously with Hs1a-FL alone (4 nmol, 45 μM of Hs1a-FL in 100 μL of PBS) or in combination with an excess of unmodified peptide (120 μM, 12 nmol in 100 μL PBS, blocking solution) and sacrificed 30 min after injection. The RSN and LSN were resected and epifluorescence imaging performed using an IVIS Spectrum imaging system (excitation = 710/45 nm, emission = 800–820 nm). In mice receiving just the imaging agent, we observed accumulation of Hs1a-FL in the resected sciatic nerves, which were clearly visible (Fig. 4a and Fig. S1c), whereas uptake was significantly reduced in the sciatic nerves of mice that received the imaging agent in combination with excess unmodified peptide (radiant efficiency: 1.6 ± 0.3 × 10^5^ and 0.09 ± 0.03 × 10^5^ for Hs1a-FL and co-injection (blocking solution), respectively; unpaired *t* test, *p* value < 0.001, Fig. 4b). A trend towards higher fluorescence signals in the liver, kidney, brain, and spleen was also observed (radiant efficiency: 3.0 ± 2.0 × 10^7^ and 0.002 ± 0.001 × 10^7^, 1.4 ± 1.1 × 10^7^ and 0.005 ± 0.004 × 10^7^, 0.2 ± 0.1 × 10^7^ and 0.001 ± 0.0005 × 10^7^, and 0.8 ± 0.5 × 10^7^ and 0.004 ± 0.0003 × 10^7^ for organs injected with fluorescent agent and with PBS, respectively; Fig. S2a and Fig. S2b). Nerve-to-muscle values showed a favorable accumulation in Hs1a-FL treated mice, compared to block and PBS (ratio: 4.83 ± 8.73, 0.48 ± 0.61, 2.90 ± 2.76, respectively, Supplementary Table 3).

## Discussion

In the present study, we developed and characterized Hs1a-FL, a near-infrared imaging agent to target human nerves. A limitation of the present study is that, in a small rodent model, the high liver accumulation makes it difficult to discern the signal coming from Na_V_1.7-expressing nerve and unspecific liver signal. However, this limitation could be overcome in humans thanks to more favorable anatomy and nerves with larger diameters. Due to the preclinical model chosen, the sciatic nerves represent the largest peripheral nervous system target for our molecule in vivo. Hs1a is a peptide derived from the venom of a Chinese tarantula, with a low nanomolar affinity for neuronal sodium channels. Here, we showed that conjugation of Hs1a and Cy7.5 dye yields a fluorescent tracer for nerve imaging. The synthesis of Hs1a-FL was straightforward and efficient. We evaluated Hs1a-FL for ex vivo imaging of resected sciatic nerves via microscopy as well as imaging of exposed nerves via epifluorescence. The chosen mouse model for the in vivo investigation was athymic nude mice for their versatility and ease of use. Furthermore, nude mice do not have a furred skin and therefore allow an easier surgical exposure of the nerves without the need of hair removal. The mouse model should not affect the efficacy of Hs1a-FL in imaging nerves. Hs1a-FL was shown to specifically bind to Na_V_1.7 channels in vivo post-intravenous injection, and its binding could be blocked by competition with an excess dose of the unlabeled Hs1a. A more specific nerve agent could improve imaging potential and significantly limit background noise and possible toxicity. It will be interesting to investigate in the future whether blood pools or coagulated blood could represent a limitation in the signal-to-background ratio in the operation room and to identify potential changes in the binding affinity against different Na_V_ channels after different dyes conjugation. The near-infrared emission spectrum of Cy7.5 may enable the use of this agent for visualization of nerves that are beneath the tissue surface during surgical interventions. The nerve-to-muscle ratio was calculated and showed a blockable signal of ~ 4 for Hs1a-FL-injected mice. A broader investigation is needed to identify the most ideal nerve imaging agent for intraoperative applications. This study represents the scientific basis for the development of a set of nerve-targeting agents derived from naturally available peptides.

## Conclusion

Hs1a-FL proved to be a useful tool for nerve visualization. This nerve-targeting agent should be explored further with a view to developing tools that can assist surgeons to identify peripheral nerves and avoid surgical morbidity due to nerve injury.

## Supplementary information

**Additional file 1.** Fluorescence labeling of a Na_V_1.7-targeted peptide for near-infrared nerve visualization.

## Data Availability

All data and material are made available.
