# Supplementary material for: Fluorescence labeling of a NaV1.7-targeted peptide for near-infrared nerve visualization
Source: EJNMMI Res. 2020 May 14;10:49. doi: 10.1186/s13550-020-00630-4 (PMC7225226; doi:10.1186/s13550-020-00630-4)
Supplement: Supplementary file 1 — Additional file 1. Fluorescence labeling of a NaV1.7-targeted peptide for near-infrared nerve visualization. [file 13550_2020_630_MOESM1_ESM.docx]

**SUPPLEMENTARY MATERIAL**

**Fluorescence labeling of a Na_V_1.7-targeted peptide for near-infrared nerve visualization**

Junior Gonzales^1,†^, Giacomo Pirovano^1,†^, Chun Yuen Chow^2^, Paula Demetrio de Souza Franca^1^, Lukas M. Carter^1^, Julie K. Klint^2,3^, Navjot Guru^1^, Jason S. Lewis^1,5,6,7^, Glenn F. King^2^ and Thomas Reiner^1,4,5,8,*^

^1^ Department of Radiology, Memorial Sloan Kettering Cancer Center, 1275 York Avenue, New York, New York, 10065, USA

^2^ Institute for Molecular Bioscience, The University of Queensland, St. Lucia, Queensland 4072, Australia.

^3^ Current address: H. Lundbeck A/S, Ottiliavej 9, 2500 Valby, Denmark

^4^ Center for Molecular Imaging and Nanotechnology (CMINT), Memorial Sloan Kettering Cancer Center, New York, NY, 10065, USA

^5^ Department of Radiology, Weill Cornell Medical College, 1300 York Avenue, New York, New York, 10065, USA

^6^ Molecular Pharmacology Program, Memorial Sloan Kettering Cancer Center, New York, NY, 10065, USA

^7^ Department of Pharmacology, Weill-Cornell Medical College, New York, NY, 10065, USA

^8^ Chemical Biology Program, Memorial Sloan Kettering Cancer Center, 1275 York Avenue, New York, New York, 10065, USA

Running title: **Labeling of a Na_v_1.7-targeted peptide**

Category: Molecular neuroscience; Peptides

^†^Co-first authors

***Corresponding author:**

Thomas Reiner, Ph.D.

Department of Radiology, MSK

1275 York Avenue

New York, NY 10065

Phone: 646-888-3461

Email: reinert@mskcc.org

**Table 1.** Hs1a selectivity for Na_v_ channels stably expressed on the membranes of HEK293 cells.

| Hs1a selectivity for Na_v_ channels stably expressed on the membranes of HEK293 cells | | | |
| --- | --- | --- | --- |
| **Ion channel** | **sub-type** | **IC_50_** | **feature** |
| **Na_v_** | Na_v_1.1 | 19.4 nM | ganglia |
|  | Na_v_1.2 | 82.2 nM | unmyelinated neurons, ganglia |
|  | Na_v_1.3 | 106.8 nM | mostly fetal nervous system |
|  | Na_v_1.4 | >3000 | adult neuro-muscular junction |
|  | Na_v_1.5 | >3000 | developing SM and cardiac muscle |
|  | Na_v_1.6 | 168 nM | axons |
|  | Na_v_1.7 | 45.7 nM | axons |

**Table 2.** Details of Hs1a and Hs1a-FL

| Details of Hs1a and Hs1a-FL | | | | |
| --- | --- | --- | --- | --- |
| **NAME** | **AMINO ACID SEQUENCE** | **LENGTH (aa)** | **Dye** | **mw (kDa)** |
| **Hs1a** | GNDCLGFWSACNPKNDKCCANLVCSSKHKWCKGKL | 35 | None | 3850.74 |
| **hs1a-fl** | Cy7.5@GNDCLGFWSACNPKNDKCCANLVCSSKHKWCKGKL | 35 | Cy7.5 | 4482.12 |

**Table 3.** Details of Hs1a-FL nerve-to-muscle comparison

| Nerve-to-muscle comaprison | | | | |
| --- | --- | --- | --- | --- |
| **PBS** | **2.90 ± 2.76** |  |  |  |
| **Block** | **0.48 ± 0.61** | 35 | None | 3850.74 |
| **hs1a-fl** | **4.83 ± 8.73** | 35 | Cy7.5 | 4482.12 |

**Supplementary Figures**





**Figure S1.** (**a**) RP-HPLC chromatogram of Hs1a-FL with detection at 780 nm. (**b**) Immunohistochemical IgG control of a mouse sciatic nerve. (**c**) Epifluorescence images of sciatic nerves from animals injected with PBS, Hs1a-FL (4 nmol, 45 µM of Hs1a-FL in 100 µL of PBS) or a mixture of unlabeled Hs1a and labeled Hs1a-FL (Hs1a-FL, 45 µM, 4 nmol and Hs1a 120 µM, 12 nmol in 100 µL PBS). Images were taken 30 min after tail vein injection.





**Figure S2.** (**a**) Epifluorescence images of whole biodistribution in animals injected with PBS, Hs1a-FL (4 nmol, 45 µM of Hs1a-FL in 100 µL of PBS) or Hs1a/Hs1a-FL (Hs1a-FL, 45 µM, 4 nmol and Hs1a 120 µM, 12 nmol in 100 µL PBS). Images were taken 30 min after tail vein injection. (**b**) Biodistribution quantification. Unpaired *t*-test was used to calculate statistical significance. **p*-value < 0.05, ***p*-value < 0.01. Error bars show standard deviations.
